# Cannabidiol Improves Antioxidant Capacity and Reduces Inflammation in the Lungs of Rats with Monocrotaline-Induced Pulmonary Hypertension

**DOI:** 10.3390/molecules27103327

**Published:** 2022-05-22

**Authors:** Anna Krzyżewska, Marta Baranowska-Kuczko, Anna Jastrząb, Irena Kasacka, Hanna Kozłowska

**Affiliations:** 1 Department of Experimental Physiology and Pathophysiology, Medical University of Bialystok, Mickiewicz 2A, 15-222 Bialystok, Poland; marta.baranowska@umb.edu.pl (M.B.-K.); hkozl@umb.edu.pl (H.K.); 2 Department of Clinical Pharmacy, Medical University of Bialystok, Mickiewicz 2A, 15-222 Bialystok, Poland; 3 Department of Analytical Chemistry, Medical University of Bialystok, Mickiewicz 2D, 15-222 Bialystok, Poland; anna.jastrzab@umb.edu.pl; 4 Department of Histology and Cytophysiology, Medical University of Bialystok, Mickiewicz 2C, 15-222 Bialystok, Poland; kasacka@umb.edu.pl

**Keywords:** pulmonary hypertension, cannabidiol, oxidative stress, inflammation, monocrotaline

## Abstract

Cannabidiol (CBD) is a plant-derived compound with antioxidant and anti-inflammatory properties. Pulmonary hypertension (PH) is still an incurable disease. CBD has been suggested to ameliorate monocrotaline (MCT)-induced PH, including reduction in right ventricular systolic pressure (RVSP), a vasorelaxant effect on pulmonary arteries and a decrease in the white blood cell count. The aim of our study was to investigate the effect of chronic administration of CBD (10 mg/kg daily for 21 days) on the parameters of oxidative stress and inflammation in the lungs of rats with MCT-induced PH. In MCT-induced PH, we found a decrease in total antioxidant capacity (TAC) and glutathione level (GSH), an increase in inflammatory parameters, e.g., tumor necrosis factor alpha (TNF-α), interleukin-1β (IL-1β), nuclear factor kappa B (NF-κB), monocyte chemoattractant protein-1 (MCP-1), and cluster of differentiation 68 (CD68), and the overexpression of cannabinoid receptors type 1 and 2 (CB_1_-Rs, CB_2_-Rs). Administration of CBD increased TAC and GSH concentrations, glutathione reductase (GSR) activity, and decreased CB_1_-Rs expression and levels of inflammatory mediators such as TNF-α, IL -1β, NF-κB, MCP-1 and CD68. In conclusion, CBD has antioxidant and anti-inflammatory effects in MCT-induced PH. CBD may act as an adjuvant therapy for PH, but further detailed preclinical and clinical studies are recommended to confirm our promising results.

## 1. Introduction

Pulmonary hypertension (PH) is an incurable disease with rapid progress and poor prognosis. PH is defined as when the mean value of pulmonary artery pressure measured by right ventricular catheterization at rest is over 25 mmHg, although it has been recently suggested to reduce this value to 20 mmHg [1]. The pathogenesis of PH is complex, and includes dysfunction of the vascular endothelium accompanied by excessive vasoconstriction, increased oxidative stress, enhanced inflammation, remodeling of pulmonary arteries with intimal hypertrophy, and right heart failure, which in consequence leads to premature death. Increased oxidative stress and inflammation induce the remodeling of pulmonary vessels, and participate in vasoconstriction in addition to an increase in pulmonary vascular resistance [1,2]. In human PH, perivascular inflammation is associated with an increase in inflammatory mediators (tumor necrosis factor alpha (TNF-α), interleukin-1β (IL-1β), cluster of differentiation 68 (CD68) and nuclear factor kappa B (NF-κB)), in addition to the accumulation and infiltration of inflammatory cells, e.g., macrophages which correlates with disease progression [3,4]. According to the reports that combination therapy reduces the risk of clinical deterioration of patients, the European Society of Cardiology (ESC) and the European Respiratory Society (ERS) recommend polypharmacology, i.e., use of drugs (or combinations of drugs) that have multiple effects, for PH treatment [5]. Thus, drugs with additional target points, including antioxidant and anti-inflammatory effects are recommended, in order to have a pleiotropic effect on the pathogenesis of PH. The available therapies for PH only improve life quality of patients, but do not guarantee a full recovery, which underscores the need for novel therapeutic strategies [1].

Cannabidiol (CBD) is a non-intoxicating compound isolated from *Cannabis sativa var. indica*. CBD has pleiotropic properties, including those which are antioxidant and anti-inflammatory [6,7]. In studies carried out on the mouse model of oxygen-glucose deprivation/reperfusion injury, it was found that CBD supplementation reduced the level of malondialdehyde, which is a product of lipid peroxidation. Chronic administration of CBD to rats and mice reduced the number of oxidative stress markers, causing a pronounced increase in glutathione (GSH) levels in both animal models—for a review, see Atalay [7]. CBD reduced proinflammatory cytokines in lung tissue in a mouse asthma model [8], and in mouse lipopolysaccharide (LPS)-induced acute lung injuries [9].

CBD is believed to have a weak affinity for cannabinoid receptors type 1 and 2 (CB_1_-R and CB_2_-R), but may also act indirectly by regulating the levels of certain endocannabinoids [6]. Additionally, CBD is an allosteric negative modulator of the CB_1_-R, which means that it could reduce the potency of CB_1_-R ligands, thus limiting the adverse effects associated with the activation of CB_1_-Rs [6]. Moreover, CBD is able to antagonize the effects of the CB_1_-R agonist (CP55940) at nanomolar concentrations, i.e., lower than that resulting from its affinity for these receptors. Additionally, the authors suggest that this antagonism is non-competitive in nature [10]. Moreover, activation of CB_1_-Rs has been shown to increase proinflammatory signaling and evoke oxidative stress [11]. Therefore, blocking CB_1_-Rs would result in reduction in inflammation and fibrosis in mouse lungs [11]. Activation of CB_2_-Rs produces opposite effects, i.e., reduces lung inflammatory damage and pulmonary edema [12], and may be a promising target point in inhibition of inflammation in COVID-19 [11].

Cannabinoids, including CBD, have potentially beneficial effects in PH [13]. CBD has been suggested as an adjuvant therapy for the treatment of PH as a result of its relaxing effect in human pulmonary arteries [14]. In addition, CBD reduced right ventricular systolic pressure (RVSP) and pulmonary vascular hypertrophy in both the rat model of PH induced by monocrotaline (MCT) [15,16], and in the Sugen-hypoxia PH mouse model [16], which suggest that CBD may be able to alleviate PH.

Due to the current trend of using polypharmacological approaches in PH treatment, together with the proven beneficial effects of CBD in various PH models as a consequence of its antioxidant and anti-inflammatory properties, the aim of the study was to evaluate the effect of CBD on the selected parameters of oxidative stress and inflammation in the lungs of rats with MCT-induced PH. Although previous studies suggest that CBD may have anti-inflammatory potential in the mouse model of Sugen-hypoxia-induced PH [16], it has been recently reported that the Sugen-hypoxia model is not preferable in mice, since they display very modest vascular remodeling compared to rats [17,18,19]. In this respect, both pulmonary vascular remodeling and PH are reversible, and no characteristic perivascular infiltration of monocytes/macrophages is observed [17,18,19]. The MCT-induced PH model in rats is more recommended because it particularly shows the role of inflammation in the development of PH, and allows the development of damage to pulmonary vessel endothelia with the characteristic perivasculitis [18].

Altogether, we have shown for the first time that CBD improved antioxidant capacity and reduced inflammation in the rat model of MCT-induced PH.

## 2. Results

### 2.1. Influence of PH and Chronic Administration of CBD on RVSP

As described previously [15], the RVSP was higher in the MCT group (43.7 ± 3.9 mmHg, *n* = 10) compared to the control (CTR) group (20.03 ± 0.9 mmHg, *n* = 10; *p* < 0.001). Chronic CBD administration reduced the RVSP in the MCT + CBD group (28.2 ± 0.7 mmHg, *n* = 10, *p* < 0.001) compared to MCT group but not in the control group (CTR + CBD 21.4 ± 0.9 mmHg, *n* = 10).

### 2.2. Influence of PH and Chronic Administration of CBD on Oxidative Stress in Lung Tissue

**Figure 1:**
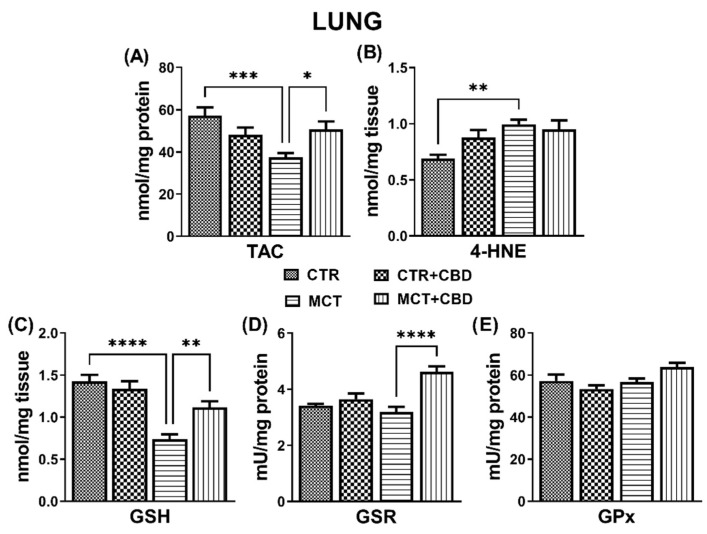
The influence of monocrotaline (MCT)-induced pulmonary hypertension and cannabidiol (CBD) or its vehicle on redox balance parameters in rats’ lungs. Total antioxidant capacity, TAC (**A**); 4-hydroxyhexenal, 4-HNE (**B**); reduced glutathione, GSH (**C**); glutathione-disulfide reductase, GSR (**D**); glutathione peroxidase, GPx (**E**). CBD 10 mg/kg or its vehicle were injected *i.p.* every 24 h for 21 days. Data are presented as mean ± SEM, (*n* = 5–7 per group); * *p* < 0.05, ** *p* < 0.01, *** *p* < 0.001 and **** *p* < 0.0001, compared to the respective group.

MCT administration reduced total antioxidant capacity (TAC) (−35%) (Figure 1A) and concentration of GSH (−48%) (Figure 1C) in the lung tissues compared to the CTR group. MCT increased the concentration of 4-hydroxyhexenal (4-HNE) (+44%), which is a product of lipid peroxidation, compared to CTR (Figure 1B). However, MCT-treated rats did not modify the activity of antioxidant enzymes, such as glutathione-disulfide reductase (GSR) and glutathione peroxidase (GPx) (Figure 1D and Figure 1E, respectively).

CBD administration to MCT-treated rats increased concentrations in antioxidant compounds TAC (+36%) (Figure 1A) and GSH (+51%) (Figure 1C). CBD also increased the enzymatic activity of GSR (+45%) (Figure 1D) in the MCT + CBD group compared to MCT group. Administration of CBD did not change the MCT-stimulated increased concentration in 4-HNE (Figure 1B), and did not change GPx activity (Figure 1E). No changes of the above-mentioned parameters were observed in the lungs of CTR rats receiving CBD (CTR + CBD) (Figure 1).

### 2.3. Influence of PH and Chronic Administration of CBD on Inflammation Parameters in Lung Tissue

**Figure 2:**
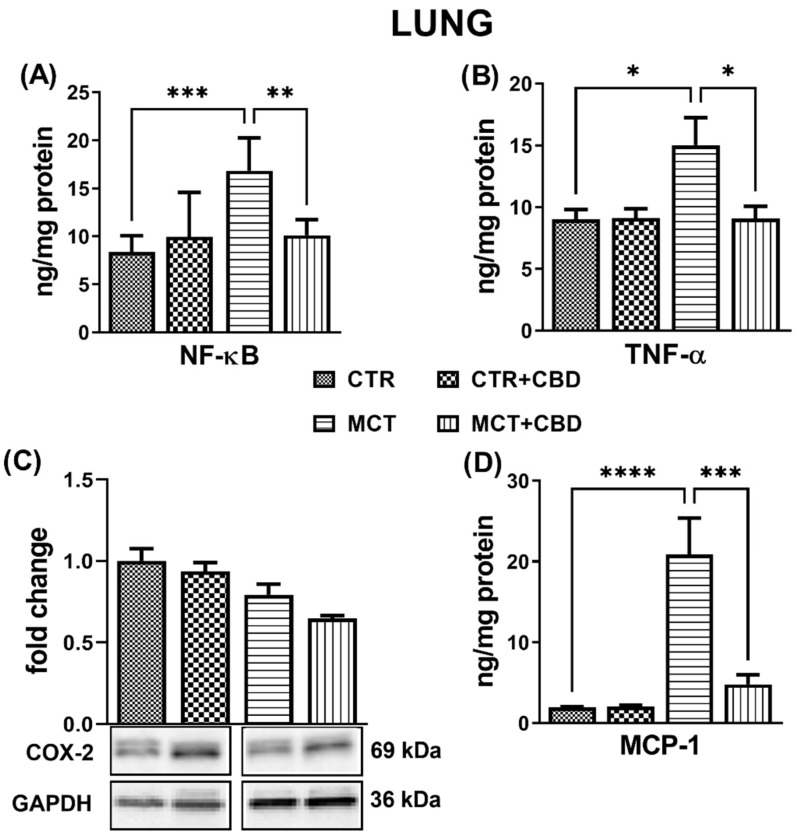
The influence of monocrotaline (MCT)-induced pulmonary hypertension and cannabidiol (CBD) or its vehicle on parameters of inflammation in rats’ lungs. Nuclear factor kappa B, NF-κB (p65) (**A**); tumour necrosis factor alpha, TNF-α (**B**); cyclooxygenase 2, COX-2 (**C**); monocyte chemoattractant protein-1, MCP-1 (**D**). Bar graph C illustrates the fold changes (for the relative fold change in expression in comparison to the respective CTR, whose expression level was set to 1) in the density of COX-2. CBD 10 mg/kg or its vehicle were injected *i.p.* every 24 h for 21 days. Data are presented as mean ± SEM, (*n* = 6–7 per group); * *p* < 0.05, ** *p* < 0.01, *** *p* < 0.001 and **** *p* < 0.0001, compared to the respective group.

The concentrations of inflammatory parameters NF-κB, TNF-α and monocyte chemoattractant protein-1 (MCP-1) in lung tissues were analysed using an ELISA method. MCT administration increased the concentrations in NF-κB (+100%) (Figure 2A), TNF-α (+67%) (Figure 2B), and MCP-1 (+10-fold) (Figure 2D) in the lung tissues compared to CTR rats.

Chronic administration of CBD to MCT-treated rats decreased the concentrations in NF-κB (−40%) (Figure 2A), TNF-α (−40%) (Figure 2B), and MCP-1 (−77%) (Figure 2D). No changes were observed when CBD was administered to CTR rats (Figure 2). The densities of cyclooxygenase 2 (COX-2) evaluated by Western blot method were similar in each group (Figure 2C).

**Figure 3:**
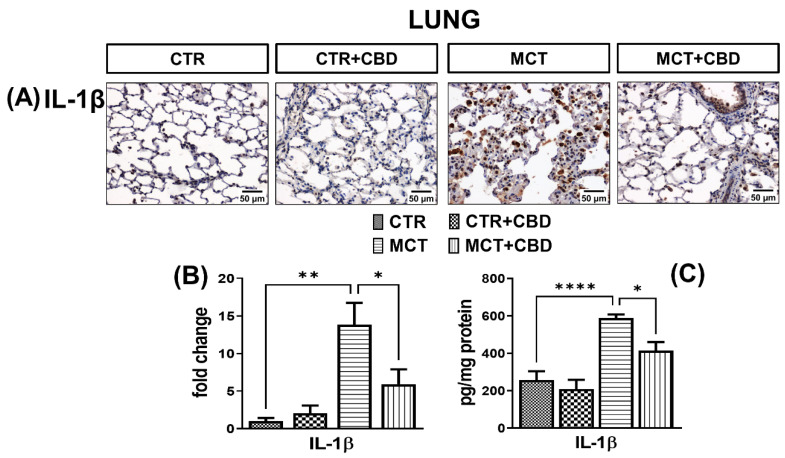
The influence of monocrotaline (MCT)-induced pulmonary hypertension and cannabidiol (CBD) or its vehicle on interleukin-1β (IL-1β) (**A**); representative micrographs of immunohistochemical staining (magnification 200×) (**B**) and their quantification; (**C**) the concentration of IL-1β determined by enzyme-linked immunosorbent assay (ELISA) in rats’ lungs. Bar graphs (**B**,**C**) illustrate the fold changes (for the relative fold change in expression in comparison to the respective CTR, whose expression level was set to 1) in the percentage area stained for IL-1β and concentration of IL-1β, respectively. The dark brown precipitate represents the intensity of IL-1β. CBD (10 mg/kg) or its vehicle was injected *i.p*. every 24 h for 21 days. Data are presented as mean ± SEM, (*n* = 6–7 per group); * *p* < 0.05, ** *p* < 0.01, and **** *p* < 0.0001, compared to the respective group.

Positive immunohistochemical reaction demonstrated that IL-1β was found mainly in lung macrophages and lung interstitial cells (Figure 3A). A much stronger immunoreactivity of IL-1β (by about 13-fold) was demonstrated in the lungs of MCT rats (Figure 3A,B). MCT administration increased the concentration in IL-1β by about 128% evaluated by an ELISA method (Figure 3C).

Chronic administration of CBD to MCT-treated rats reduced IL-1β immunoreaction intensity by about 57% (Figure 3A,B), and its concentrations by about 30% (Figure 3C). No differences were observed in immunoreactivity or in concentrations of IL-1β in the lungs of CTR animals receiving CBD (Figure 3).

**Figure 4:**
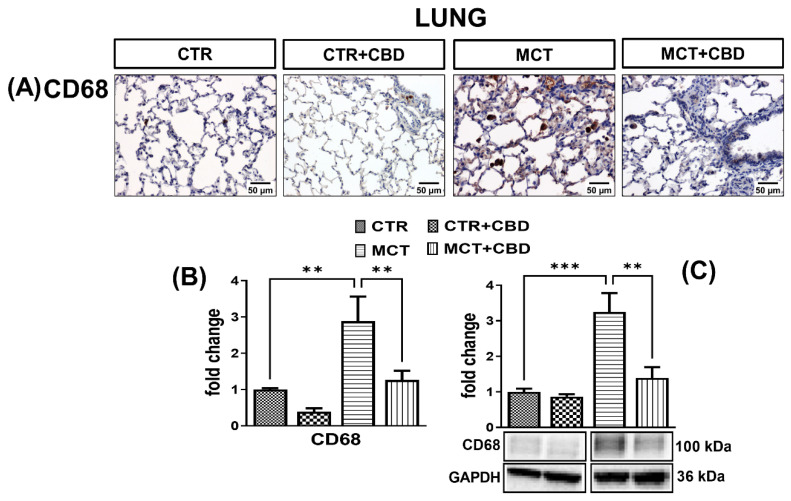
The influence of monocrotaline (MCT)-induced pulmonary hypertension and cannabidiol (CBD) or its vehicle on cluster of differentiation (CD68) (**A**); representative micrographs of immunohistochemical staining (magnification 200×) (**B**) and their quantification; and (**C**) the density of CD68 determined by Western blot in rats’ lungs. Bar graphs B and C illustrate the fold changes (for the relative fold change in expression in comparison to the respective CTR, whose expression level was set to 1) in the percentage area stained for CD68 and in the density of CD68, respectively. The dark brown precipitate represents the intensity of CD68. CBD (10 mg/kg) or its vehicle was injected *i.p.* every 24 h for 21 days. Data are presented as mean ± SEM, (*n* = 6–7 per group); ** *p* < 0.01, *** *p* < 0.001, compared to the respective group.

The immunohistochemical reaction using the anti-CD68 antibody showed a greater number of macrophages, with an approximately two-fold greater immunoreactivity in the lungs of MCT rats compared to CTR (Figure 4A,B). The density of CD68 analysed by Western blot method was increased in the lungs of MCT rats by about two-fold (Figure 4C).

Administration of CBD to MCT-treated rats decreased the number of CD68-immunopositive cells (Figure 4A), the intensity of the immunohistochemical reaction (Figure 4B), and the density (Figure 4C) of CD68 by about 56% in both cases. In the lungs of CTR and CTR + CBD rats, weak CD68 immunoreactivity was observed only in single cells (Figure 4A).

### 2.4. Influences of PH and Chronic Administration of CBD on Expression of Cannabinoid Receptors in Lung Tissue

**Figure 5:**
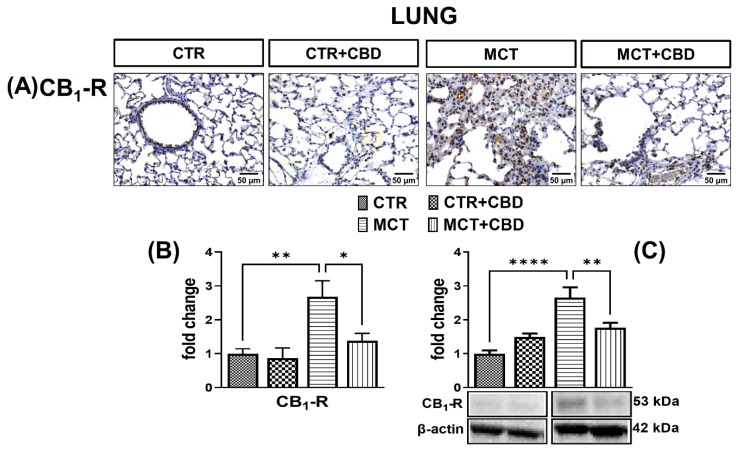
The influence of monocrotaline (MCT)-induced pulmonary hypertension and cannabidiol (CBD) or its vehicle on cannabinoid receptor type 1 (CB_1_-R) (**A**); representative micrographs of immunohistochemical staining (magnification 200×) (**B**) and their quantification; and (**C**) the density of CB_1_-R determined by Western blot in rats’ lungs. Bar graphs B and C illustrate the fold changes (for the relative fold change in expression in comparison to the respective CTR, whose expression level was set to 1) in the percentage area stained for CB_1_-R and in the density of CB_1_-Rs, respectively. The dark brown precipitate represents the intensity of CB_1_-R. CBD (10 mg/kg) or its vehicle was injected *i.p.* every 24 h for 21 days. Data are presented as mean ± SEM, (*n* = 6–7 per group); * *p* < 0.05, ** *p* < 0.01 and **** *p* < 0.0001, compared to the respective group.

**Figure 6:**
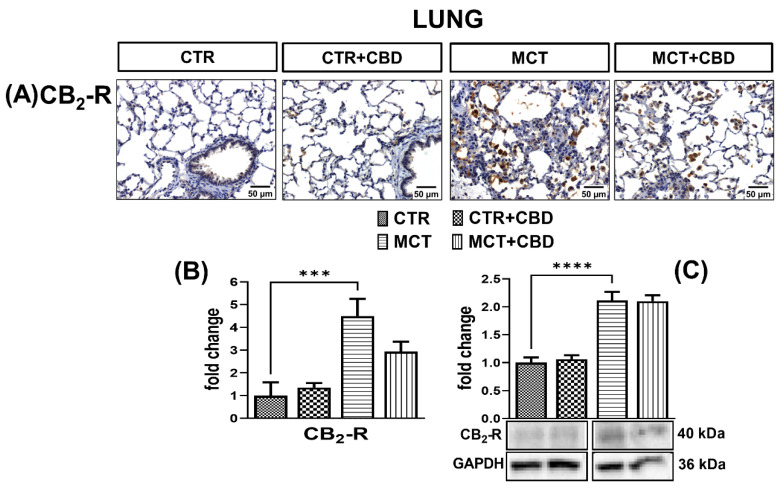
The influence of monocrotaline (MCT)-induced pulmonary hypertension and cannabidiol (CBD) or its vehicle on cannabinoid receptor type 2 (CB_2_-R) (**A**); representative micrographs of immunohistochemical staining (magnification 200×) (**B**) and their quantification; and (**C**) the density of CB_2_-R determined by Western blot in rats’ lungs. Bar graphs B and C illustrate the fold changes (for the relative fold change in expression in comparison to the respective CTR, whose expression level was set to 1) in the percentage area stained for CB_2_-R and in the density of CB_2_-Rs, respectively. The dark brown precipitate represents the intensity of CB_2_-R. CBD (10 mg/kg), or its vehicle was injected *i.p*. every 24 h for 21 days. Data are presented as mean ± SEM, (*n* = 6–7 per group); *** *p* < 0.001 and **** *p* < 0.0001, compared to the respective group.

CB_1_-R (Figure 5A,B) and CB_2_-R (Figure 6A,B) immunostaining in the lungs of MCT rats results in a strong response in the cells. The densities of CB_1_-Rs (Figure 5C) and CB_2_-Rs (Figure 6C) determined by Western blot method were increased (by about 160% and 100%, respectively) in the lungs of MCT rats.

Administration of CBD to MCT-treated rats decreased the immunoreactivity (Figure 5A,B) and density (Figure 5C) in CB_1_-Rs by about 50% and 34%, respectively, but CB_2_-R expression was unchanged in both cases (Figure 6A–C). After administration of CBD to MCT rats, we only observed a downward trend of CB_2_-Rs expression (Figure 6B). No changes of these receptors were observed in CTR rats treated with CBD (Figure 5 and Figure 6).

## 3. Discussion

The aim of the study was to evaluate whether chronic administration of CBD had a beneficial effect on the parameters of oxidative stress and inflammation in rats with MCT-induced PH. In addition, we present for the first time how MCT-induced PH affects cannabinoid receptors (CB_1_-R and CB_2_-R) in rat lung tissues, and how chronic CBD administration regulates the expressions of these receptors.

The experiments were performed on lung tissue from animals with MCT-induced PH. We used this model because it well reflects changes in human’s PH, and causes selective damage to pulmonary circulation. The MCT model is an inflammatory model that has been used in experimental studies with drugs already used in the standard treatment of PH [18].

We administered CBD at a dose of 10 mg/kg for 21 days *i.p.* This dose was established based on literature data, where chronic administration of CBD has shown positive effects; it was calculated that this dose corresponds to a dose of 800 mg for a person weighing 80 kg [20]. CBD in the same dose reduced RVSP, pulmonary arterial and right ventricular hypertrophy [15,16]. Chronic administration of CBD, at a dose of 10 mg/kg modified the levels of oxidative stress parameters, changed the expression of CB-Rs in the hearts and plasma [21], and improved vasodilation in aortas and small mesenteric arteries [22] in rats with primary and secondary hypertension. Additionally, in the mouse autoimmune myocarditis model, the administration of CBD at a dose of 10 mg/kg for 46 days *i.p.* improved the contractile function of the heart and furthermore reduced inflammation [23].

### 3.1. Influence of PH and Chronic CBD Administration on Antioxidant Status

Redox imbalance to the detriment of antioxidant substances is one of the features of PH. Oxidative stress contributes mainly to the dysfunction of the pulmonary endothelium, which results in increased resistance in pulmonary circulation. Excessive production of reactive oxygen species (ROS) contributes to the reduced nitric oxide bioavailability, and thus intensifies the contraction and remodeling of pulmonary vessels [24]. In our study, we observed reduced levels of total antioxidant capacity (TAC) and GSH in the lung tissue of rats with PH. Glutathione is a powerful antioxidant that is involved in regulating the content of reactive nitrogen species (RNS) and ROS. Excess ROS and RNS can damage proteins and lipids [25]. We also observed an increased concentration in 4-HNE in the lungs of rats with PH, which is the main product of lipid peroxidation resulting from increased oxidative stress. The increase in 4-HNE may be related to a decrease in GSH, since under normal conditions 4-HNE is neutralized by glutathione S-transferase which conjugates 4-HNE to GSH [26]; with reduced GSH levels, neutralization of 4-HNE may become less efficient. In the plasma of rats with MCT-induced PH, an increased development in oxidative stress and lipid peroxidation products is observed [27]. Importantly, ROS levels are three times higher in plasma from patients with PH [28], and the mitochondria and nicotinamide adenine dinucleotide phosphate (NADPH) are responsible for the production of ROS in cardiovascular disorders including PH [24].

Reduced antioxidant activity is closely related to the pathogenesis of PH, and CBD is a compound with antioxidant potential (see Introduction). In our study, we observed an increase in TAC after chronic administration of CBD. Similarly, GSH concentration and GSR activity increased after CBD administration. The role of GSR is to maintain an appropriate concentration of GSH; therefore, CBD probably increases the activity of the GSR enzyme, also increasing the level in GSH. Our results are consistent with previous reports of a particularly pronounced increase in GSH levels in the myocardial tissues of mice with diabetic cardiomyopathy after CBD administration [29]. Chronic administration of CBD in the same dose (10 mg/kg) increased the levels in GSH in the plasma and heart of rats with primary and secondary hypertension, and increased the level in vitamin E in the plasma of rats with secondary hypertension [21]. Additionally, CBD recovered the dysfunctional mitochondria in hypoxia condition and reduced oxidative stress and excessive glycolysis induced by hypoxia in human PH-PASMC cell culture [16]. CBD is also known to support the action of antioxidant enzymes by preventing the depletion of elements such as zinc and selenium, which are usually lowered in pathological conditions. These elements are essential for the biological activity of antioxidant enzymes. In addition, CBD, by lowering the levels in ROS, prevents the oxidation of GSH and other non-enzymatic antioxidants [7].

### 3.2. Influence of PH and Chronic CBD Administration on Inflammation

Higher levels of proinflammatory cytokines in lung tissue and/or plasma have been confirmed in human PH and in PH animal models [4]. It has also been suggested that the uncontrolled secretion of inflammatory mediators is one of the factors enhancing lung remodeling in human PH [30]. In our study, there seems to be a relationship between the NF-κB pathway, and the inflammatory condition associated with PH. The increase in NF-κB concentration in the lung tissue of rats with PH is associated with an increase in proinflammatory cytokines, i.e., TNF-α and IL-1β, and an increase in MCP-1 is also noticeable. NF-κB is known to be a central regulator of the genes responsible for the development of inflammation in PH [31]. As mentioned above, ROS promote the development of PH by activation of the NF-κB pathway and the activator protein 1 (AP-1), thus stimulating the growth of MCP-1, which is responsible for the infiltration of monocytes and macrophages. Infiltrating inflammatory cells increases the secretion of inflammatory cytokines TNF-α and IL-1β [32]. Our results are consistent with previous reports in which rats with hypoxia-induced PH showed an increase in NF-κB, IL-1β and TNF-α parameters [33].

We confirmed the increased infiltration of monocytes and macrophages using two independent methods. Activated CD68-labeled macrophages play an important role in remodeling pulmonary vessels. Macrophages are a source of leukotriene B4 (LTB4), which is responsible for damaging the endothelium of the pulmonary arteries and consequently, increasing the proliferation and hypertrophy of smooth muscle cells. LTB4 blockade is effective in improving pulmonary vascular function, and a reduction in CD68 macrophages reduces the development of SU5416-induced PH in rats [34]. In our immunohistochemistry study we demonstrated increased infiltration of macrophages in the perivascular spaces. We observed that the alveolar macrophages of the MCT rats showed morphological signs of activation, such as enlargement and growth of intracellular vacuoles predominantly in lung interstitial tissue. Thus, we confirmed the results of Xu et al. [35] who, in addition to showing increased expression in CD68 in the lung tissue of rats with MCT-induced PH, showed that macrophage infiltration increased with the development of PH and was the highest on the 28th day of the experiment.

We found no differences in COX-2 expression in the CTR rats and MCT-treated rats. Our results are closest to those reported by Seta et al. [36], who demonstrated no differences in the rats’ lung tissue between CTR and MCT-induced PH. Literature data on COX-2 expression are unclear, since cyclooxygenase 1 (COX-1) and COX-2 were strongly overexpressed in human lung tissues [37]; in the lungs of hypoxic-induced PH rats, an increased expression in COX-2 with unchanged COX-1 expression was detected [38]. Supposedly, this may be due to the differences in models used in the experiments, as well as the length of induction of PH (in humans, the development of PH takes many years).

Excessive inflammation mediates further stages of pulmonary vascular remodeling in human PH and animal PH models, as discussed in more detail in Rabinovitch et al. [34]. CBD is a compound with anti-inflammatory properties, but the mechanisms of action are not fully understood [6]. In our preventive model, CBD reduced the concentrations in TNF-α and IL-1β, which are dependent on the NF-κB pathway, as well as MCP-1 and CD68, which are associated with macrophage infiltration. A higher lung TNF-α level determined by mRNA expression was shown in the Sugen-hypoxia mouse model [16], however, as mRNA levels do not always reflect protein levels [39], we confirmed these results based on the protein level in the lungs of rats with MCT-induced PH. Our results confirm that chronic administration of CBD down-regulates inflammation, and further suggest that the anti-inflammatory properties of CBD are related to the NF-κB pathway. Moreover, the NF-κB pathway also participates in the activation of MCP-1 genes. In our research, the decrease in the concentration of NF-κB after chronic administration of CBD was correlated with a decrease in the concentration of MCP-1 in lung tissues. Presumably, inhibition of the NF-κB pathway by CBD is associated with reduced infiltration of monocytes and macrophages into the perivascular tissue of the lungs, limiting the development of inflammation, and therefore the unfavourable remodeling of pulmonary vessels. The above-mentioned notion is also confirmed by our analysis, where CBD decreased CD68 expression which is widely expressed in macrophages. Kozela et al. [40] showed that CBD reduced the levels of proinflammatory IL-1β and IL-6 in LPS-activated BV-2 microglial cells, and that this reduction was not dependent on CB_1_-Rs/CB_2_-Rs. The authors suggested that CBD can reduce the level of cytokines, mainly IL-1β and IL-6, via the NF-κB-dependent pathway by inhibiting the phosphorylation of the NF-κB p65 subunit [40]. CBD also reduced TNF-α concentration and NF-κB activity in RAW 264.7 macrophages [41]. Muthumalage and Rahman [42] also observed a reduction in MCP-1 in human bronchial epithelial cells after CBD administration, and suggested that this may be related to the reduction in NF-κB activity.

The NF-κB pathway has been suggested to be associated with the regulation of the proinflammatory *COX-2* gene. We did not observe the influence of CBD on the density of COX-2 in the lungs of rats with PH; however, acute inhibition of COX-2 can be dangerous in the early stages of the disease. COX-2 is one of the mediators of the reaction in which compounds with a vasodilating effect on the pulmonary vessels are also formed (prostacyclin). Fredenburgh et al. [43] even suggested that deficiency or pharmacological blocking of COX-2 exacerbates hypoxia-induced PH in mice.

### 3.3. Influence of PH and Chronic CBD Administration on Expression of Classic Cannabinoid Receptors

In our study, we have shown for the first time the overexpression of CB_1_-Rs and CB_2_-Rs in PH. Increased CB_2_-R density in lung tissue is probably associated with the presence of numerous inflammatory cells known to express CB_2_-Rs to a great extent [6,13]. Moreover, it has been suggested that TNFα and IL-1β can directly upregulate CB_1_-Rs and CB_2_-Rs in human blood [44]. It is also noteworthy that the activation of CB_1_-Rs intensifies the proinflammatory response, especially related to TNF-α, and is associated with increased oxidative stress through increased production of ROS. On the contrary, in the case of the CB_2_-R, its activation reduces the amount of ROS and TNF-α [7]. The endocannabinoid system seems to play a role in PH, although it is not entirely clear how significant it is. In our previous work, [15] we have shown that *N*-arachidonoyl glycine (NAGLy) and palmitoleoyl ethanolamide (POEA) levels are reduced in the lung tissue of rats with MCT-induced PH, and NAGLy is an endocannabinoid with systemic vasodilating potential [13].

We have shown that CBD reduces the density of CB_1_-Rs in the lungs of rats with PH. The proinflammatory effect of CB_1_-Rs was recently confirmed by Haddad [45], where administration of arachidonyl-2′-chloroethylamide (ACEA), a selective agonist of CB_1_-Rs, caused an increase in IL-6 concentration in rat skeletal muscle myotubes. One of the mechanisms of such regulation may be the Gi/PI3K–Akt/NF-kB pathway. Notably, the activation of CB_1_-Rs participates in damage and inflammation of the lungs through IL-1β, TNF-α and MCP-1 signaling [11,46]. Thus, it appears that reduction in CB_1_-R expression is protective in PH.

In our study, CBD did not change the CB_2_-R density which was increased by PH. However, the role of this receptor in PH is still unclear. Administration of the CB_2_-R antagonist (AM630) increased IL-6 expression in mice PASMCs, but no differences in the PH phenotype were noted between the wild type PH mice and *Cnr2^−/−^* mice with PH [16].

In our study, we did not find that CBD influenced the parameters of oxidative stress and inflammation in healthy (CTR) animals. Therefore, it seems that activity of CBD might be limited to the pathological conditions, while remaining neutral in healthy animals. Overall, CBD is considered a well-tolerated and safe drug with additional pharmacological benefits, including a relaxing effect, administration by inhalation and lack of influence on systemic pressure [6], all of which make it an interesting agent to investigate in PH therapy.

### 3.4. Limitations of the Study

The present study was limited to the examination of the effects of CBD on parameters of oxidative stress and inflammation in the lung tissue of male rats. As PH is a disease that develops especially in women, it would be appropriate to extend the research to female rats [47]. Additionally, we only used one model of PH in our study. Moreover, in the future, the antioxidant and anti-inflammatory effects of CBD in lung tissue could also be extended to receptor research by administering receptor antagonists, which may modulate the effects of CBD.

## 4. Materials and Methods

### 4.1. Animals

All experimental protocols were conducted in accordance with the Local Animal Ethics Committee in Olsztyn (Poland, project code: 88/2018, approved 27 November 2018) and the European Directive (2010/63/EU). The experiments were performed following the principles of replacement, refinement or reduction. Animals were obtained from the Centre of Experimental Medicine of the Medical University of Bialystok (Bialystok, Poland). Animals were kept under a 12 h/12 h light/dark cycle and had free access to food and water. The experiments were performed on 40 male Wistar rats (5–8 weeks old, with body weights of 150–250 g).

### 4.2. Monocrotaline and CBD Treatment

Wistar rats were injected MCT with a volume of 3 mL/kg at the dose of 60 mg/kg body weight once subcutaneously (*s.c.*) on day “0”, or with vehicle for MCT at the same dose. MCT was dissolved in 1 mol/L hydrochloric acid neutralized with 1 mol/L sodium hydroxide and diluted with saline. Healthy rats as well as rats treated with MCT were injected intraperitoneally (*i.p.*) with CBD (10 mg/kg) or its vehicle (ethanol, Tween 80, 0.9% NaCl—3:1:16) every 24 h for 21 days in the following 4 groups: (1) MCT: rats treated with MCT and vehicle for CBD; (2) MCT + CBD: rats treated with MCT and CBD; (3) control (CTR): rats treated with MCT vehicle and CBD vehicle; and (4) CTR + CBD: rats treated with CBD and MCT vehicle. For the sake of simplicity, the group injected with solvents for MCT and CBD was labelled as CTR. Day 21 was selected as the endpoint of the experiments based on our preliminary study that found that rats at day 21 already had well-developed PH, and as was previously described by Chen et al. [48]. The animals were anesthetized with sodium pentobarbital (300 µmol/kg, *i.p.*). A pressure catheter (SPR-320 Mikro-Tip, Millar, Houston, TX, USA) was inserted through the right jugular vein using the closed chest method. Measurements were taken from the right ventricle and recorded on a LabChart 7.3.7 Pro (ADInstruments, Hastings, UK) [14].

### 4.3. Tissue Preparation for Biochemical and Immunohistochemistry Examinations

Rats were anesthetized with pentobarbital sodium (300 µmol/kg, *i.p.*) 24 h after the last dose of CBD or its vehicle to collect lung tissues. Following thoracotomy, the lungs and the trachea were collected in whole. After the left lung was cut off, the right lung was fixed in 10% buffered formalin by injecting a fixative with a syringe into the right main bronchus until the pleura was smooth, and fixed in formalin for 72 h at +4 °C. After fixation, the same fragments were excised from each lung and embedded in paraffin in the routine manner. Paraffin blocks were cut into 4-micrometer sections and stained with haematoxylin and eosin (H + E) for general histological evaluation. On 4-micrometer paraffin sections, immunohistochemical reactions were performed using specific antibodies. Left lungs were perfused with 0.9% saline and were snap-frozen with liquid nitrogen and stored at −80 °C for biochemical examinations.

### 4.4. Western Blot

Frozen lungs were weighed, powdered and homogenized in Mammalian Protein Extraction Reagent (MPER, Thermo Scientific, Rockford, IL, USA) that contained a cocktail of protease inhibitors (Roche Diagnostics GmbH, Mannheim, Germany). The total protein concentration was determined using the bicinchoninic acid method (Price Rapid Gold BCA, Protein Assay Kit, Thermo Scientific, Waltham, MA, USA), with bovine serum albumin as a standard. Next, homogenates were reconstituted in Laemmli buffer (Bio-Rad Laboratories, Inc., Hercules, CA, USA), separated by sodium dodecyl sulfate-polyacrylamide gel electrophoresis, transferred onto nitrocellulose membranes and blocked in EveryBlot Blocking Buffer (Bio-Rad Laboratories, Inc., Tokyo, Japan). The membranes were incubated overnight at 4°C with corresponding primary antibodies in appropriate dilutions (i.e., CB1-R (1:500; Abcam, Cambridge, UK), CB2-R (1:500; Abcam, Cambridge, UK), CD68 (1:500; Abcam, Cambridge, UK), glyceraldehyde 3-phosphate dehydrogenase (GAPDH) (1:10,000; Abcam, Cambridge, UK), cyclooxygenase 2 (COX-2) (1:500; Abcam, Cambridge, UK) and β-actin (1:3000; Abcam, Cambridge, UK)). In order to detect proteins, anti-rabbit primary and anti-goat IgG horseradish peroxidase-conjugate secondary antibodies (1:3000; Abcam, Cambridge, UK) were used. After adding a suitable substrate for horseradish peroxidase (Clarity Western ECL Substrate; Bio-Rad Laboratories, Inc., Santa Cruz, CA, USA), the protein bands were quantified densitometrically using a ChemiDoc visualization system (Image Laboratory Software Version 6.0.1; Bio-Rad, Warsaw, Poland). The levels of the protein detected were normalized to β-actin or GAPDH.

### 4.5. ELISA/Colorimetric Assays

The concentrations of MCP-1, IL-1β, TNF-α and NF-κB (p65) were measured using an enzyme-linked immunosorbent assay (ELISA). Total antioxidant capacity (TAC) was determined with the ImAnOx Kit from Immundiagnostik (Bensheim, Germany) according to the manufacturer’s protocol. The reaction is based on the elimination of exogenous hydrogen peroxide by antioxidants contained in the sample. The difference between the hydrogen peroxide added and that remaining after the reaction is proportional to the TAC. Hydrogen peroxide was measured at 450 nm absorbance. Quantification was done with the provided calibrator. All measured parameters were normalized for the concentration of total protein using the bicinchoninic acid method (Price Rapid Gold BCA, Protein Assay Kit, Thermo Scientific, Waltham, MA, USA), with bovine serum albumin as a standard.

### 4.6. Immunohistochemistry

Immunostaining was performed by the following protocol: paraffin-embedded sections were deparaffined and hydrated in pure alcohols. For antigen retrieval, the sections were subjected to pretreatment in a pressure chamber and heated using Target Retrieval Solution Citrate pH = 6.0 (Agilent Technologies, Inc. Santa Clara, CA, USA). After cooling down to room temperature, the sections were incubated with Dako REAL Peroxidase-Blocking Solution (Agilent Technologies, Inc. Santa Clara, CA, USA). The sections with the primary antibodies IL-1β (1:1000; Abcam, Cambridge, UK), CD68 (1:1000; Agilent Technologies, Inc. Santa Clara, CA, USA), CB1-R (1:1000; Abcam, Cambridge, UK) and CB2-R (1:200; Abcam, Cambridge, UK), were incubated 24 h at +4 °C in a humidified chamber. This procedure was followed by incubation with secondary antibody (EnVision FLEX, High pH (Link), HRP. Rabbit/Mouse, Agilent Technologies, Inc., Santa Clara, CA, USA). The bound antibodies were visualized by incubation with DAB Flex chromogen. The sections were finally counterstained in hematoxylin QS (Vector Laboratories, Burlingame, CA, USA), mounted and evaluated under light microscope. Sections were dehydrated, and the specificity of the antibodies was confirmed using a negative control, where the antibodies were replaced by Antibody Diluent (Vector Laboratories, Burlingame, CA, USA). The results of staining were submitted for evaluation in an Olympus BX43 microscope with Olympus DP12 camera. In each lung sample, the percentage area stained was measured using the ImageJ software version 1.53c (NIH, Bethesda, MD, USA) [49], and an average was calculated from the values obtained from the six lungs per group. In order to calculate the percentage area of staining in the lungs, the area stained was divided by the total area of lung tissue to obtain a percentage staining. The data were presented as the fold changes compared to the respective CTR group.

### 4.7. Determination of Antioxidant Enzyme Activity

The method of Mize and Langdon (previously described in [21]) was used to determine the glutathione reductase (GSR—EC.1.6.4.2) activity in lung tissue. One unit of enzyme oxidized 1 µmol of nicotinamide adenine dinucleotide phosphate (NADPH) for 1 min at 25 °C and pH 7.4. Specific enzyme activity was expressed in units per mg of protein.

Glutathione peroxidase (GPx—EC.1.11.1.9) activity was measured spectrophotometrically basing on the method of Paglia and Valentine (previously described in [21]). One unit of GPx activity was determined as the amount of enzyme catalyzing the oxidation of 1 µmol NADPH to NADP+ for 1 min at 25 °C and pH 7.4. Specific enzyme activity was expressed in units per mg of protein.

### 4.8. Determination of Non-Enzymatic Antioxidant Level

The capillary electrophoresis (CE) method (as previously described in [21]) was used to determine reduced glutathione (GSH) level. Samples were sonicated with 2 mL of a solution of ACN/H_2_O (62.5:37.5, *v*/*v*), and centrifuged at 29,620× *g* for 10 min. The separation was performed on a capillary with a total length of 50 cm (40 cm effective length) and an inner diameter of 50 µm, and was operated at 27 kV with UV detection at 200 ± 10 nm. The concentration of GSH was expressed as nmol per mg tissue.

### 4.9. Determination of Lipid Modifications

4-HNE was determined by Gas Chromatography-Tandem Mass Spectrometry (GC/MS/MS), as the O-PFBoxime-TMS derivatives (previously described in [21]). Benzaldehyde-D6 was added to the lung lysates, and aldehydes were derivatized by the addition of *O*-(2,3,4,5,6-pentafluorobenzyl)hydroxyamine hydrochloride. Samples were deproteinized by the addition of 1 mL of methanol, and OPFB-oxime aldehyde derivatives were extracted by the addition of 2 mL of hexane. The top hexane layer was transferred into borosilicate tubes and evaporated under a stream of argon gas, followed by the addition of *N*,*O*-bis(trimethylsilyl)trifluoroacetamide in 1% trimethylchlorosilane. Derivatized aldehydes were analyzed using a 7890A GC—7000 quadrupole MS/MS (Agilent Technologies, Palo Alto, CA, USA) equipped with a HP-5 ms capillary column. Derivatized aldehydes were detected in the selected ion monitoring mode. The ions used for 4-HNE-PFB-TMS identification were *m*/*z* 333.0 and 181.0. The level of 4-HNE was expressed in nmol per mg tissue.

### 4.10. Statistical Analysis

All results are expressed as the mean ± SEM of *n* animals. GraphPad Prism version 9.3.0 (GraphPad Software, San Diego, CA, USA) was used to plot the mean data. Prior to statistical analysis, all data were analysed for Gaussian distribution, then parametric tests based on validated normality tests were performed. Statistical comparisons between groups were performed using analysis of variance (ANOVA) followed by Bonferroni multiple comparison tests for all data sets. Post hoc tests were performed only when F reached the required level of statistical significance, and no significant homogeneity of variance was found. Differences were considered statistically significant if *p* < 0.05.

### 4.11. Drugs

(−)-cannabidiol (CBD) (THC-1073G-1) from THC Pharm, Frankfurt, Germany; ethanol (BA6420113) and natrium chloride (NaCl) (BA4121116) from POCH, Gliwice, Poland; Tween 80 (P1754), Crotaline (MCT; C2401-1G) and Tween 80 (P1754) from Sigma-Aldrich, Munich, Germany; MCT vehicle-1 M HCl, and the pH was adjusted to 7.4 with 1 M NaOH; pentobarbital sodium (5909991290153) from Biowet, Pulawy, Poland; Mammalian Protein Extraction Reagent (M-PER; 78501) from Thermo Scientific, Rockford, IL, USA; protease inhibitors (11836153001) from Roche Diagnostics GmbH, Mannheim, Germany; Price Rapid Gold BCA, Protein Assay Kit (A53225) from Thermo Scientific, Waltham, MA, USA; Laemmli buffer (1610737) from Bio-Rad Laboratories, Inc., Hercules, CA, USA; EveryBlot Blocking Buffer (12010020) from Bio-Rad Laboratories, Inc., Tokyo, Japan; Clarity Western ECL Substrate (102031593) from Bio-Rad Laboratories, Inc., Santa Cruz, CA, USA; CB1 (ab259323) from Abcam, Cambridge, UK; CB2 (ab3561) from Abcam, Cambridge, UK; CD68 (ab125212) from Abcam, Cambridge, UK; GAPDH (EPR16891) from Abcam, Cambridge, UK; β-actin (ab8227) from Abcam, Cambridge, UK; Goat Anti-Rabbit IgG H&L (ab6721) from Abcam, Cambridge, UK; ImAnOx (TAS/TAC) Antioxidative Capacity (KC5200) from Immundiagnostik, Bensheim, Germany; IL-1β (670.040.096) ELISA Kit from Immundiagnostik, Bensheim, Germany; TNF-α (865.000.096) ELISA Kit from Immundiagnostik, Bensheim, Germany; Nuclear Factor Kappa B ELISA Kit (SEB824Ra) from Immundiagnostik, Bensheim, Germany; Dako REAL Peroxidase-Blocking Solution (S2023) from Agilent Technologies, Inc., Santa Clara, CA, USA; IL-1β (ab9722) from Abcam, Cambridge, UK; CD68 (M0876) from Agilent Technologies, Inc., Santa Clara, CA, USA; EnVision FLEX, High pH (Link), HRP. Rabbit/Mouse (K800021-2) from Agilent Technologies, Inc., Santa Clara, CA, USA; hematoxylin QS (H-3404) from Vector Laboratories, Burlingame, CA, USA; Wash Buffer (S3006) from Agilent Technologies, Inc., Santa Clara, CA, USA; CB1-R (ab23703) from Abcam, Cambridge, UK; CB2-R (ab3561) from Abcam, Cambridge, UK.

## 5. Conclusions

The evidence presented the antioxidant and anti-inflammatory effects of CBD in the lung tissue of rats with MCT-induced PH; as well, we reported on its relaxing effect on pulmonary vessels and the properties of reducing RVSP, suggesting that CBD could be a successful adjunctive therapy in the treatment of PH. The NF-κB pathway and downregulation of CB_1_-Rs, the activation of which has pro-oxidative and proinflammatory effects, may play a special role in the antioxidant and anti-inflammatory CBD-mediated effect. The available therapies for PH treatment are focused only on vascular effects; CBD has multipotent beneficial effects, which is in line with the current trend of seeking multidirectional therapies. The promising results of our research may form the basis for a more detailed study of the effects of CBD or its derivatives on PH, especially in humans.

## Data Availability

The data presented in this study are available on request from the corresponding author.

## Sample Availability

Samples of the compounds are not available from the authors.
